# What young people want to improve their health and well-being: insights from over 1.5 million young people

**DOI:** 10.1093/heapol/czaf108

**Published:** 2026-09-30

**Authors:** Ashrita Saran, David A Ross, Renée De Jong, Howard White, David Gomez, Maria El Bizri, Rajat Khosla, Dinah Salunda, Jordan Njinowo, Merette Khalil, Etienne V Langlois, Anshu Mohan

**Affiliations:** Evaluation and Evidence Synthesis, Global Development Network, 2nd Floor, West Wing, ISID Complex, Plot No 4, 4 Vasant Kunj Institutional Area, Vasant Kunj II, Vasant Kunj, New Delhi 110070, India; Institute for Life Course Health Research, Department of Global Health, Stellenbosch University Tygerberg Campus, Francie Van Zijl Drive, 7505 Tygerberg, South Africa; Partnership for Maternal, Newborn and Child Health (PMNCH), Avenue Appia 20, 1211 Geneva 27, Switzerland; Centre for Evidence-Based Social Sciences, Lanzhou University, 222 Tianshui S Rd, Chengguan District, Lanzhou, Gansu 730000, China; Partnership for Maternal, Newborn and Child Health (PMNCH), Avenue Appia 20, 1211 Geneva 27, Switzerland; Partnership for Maternal, Newborn and Child Health (PMNCH), Avenue Appia 20, 1211 Geneva 27, Switzerland; Partnership for Maternal, Newborn and Child Health (PMNCH), Avenue Appia 20, 1211 Geneva 27, Switzerland; Lilongwe, Malawi; Adolescent's Health and Well-being DESERVE, Odza Borne 10, Yaounde, Centre Region, Cameroon; YourEgyptianDoula and White Ribbon Alliance, Advocacy and Communications, 32 Ramses St Heliopolis Cairo 11757, Egypt; Partnership for Maternal, Newborn and Child Health (PMNCH), Avenue Appia 20, 1211 Geneva 27, Switzerland; Partnership for Maternal, Newborn and Child Health (PMNCH), Avenue Appia 20, 1211 Geneva 27, Switzerland

**Keywords:** adolescent health, survey, perceptions, qualitative research, World Health Organization, youth

## Abstract

Knowing young people’s views on what they want is essential to effective policies and programmes to promote their health and well-being. The What Young People Want hybrid online/in-person survey invited young people (10–24 years) to complete the statement: ‘To improve my well-being, I want…’ Responses from the 1 510 004 respondents were analysed by content and thematic analysis using natural language processing techniques, followed by word frequency analysis to identify and quantify keywords and phrases. The respondents’ ‘wants’ were classified into five domains [good health and optimum nutrition (‘Health’); connectedness, positive values and contribution to society (‘Connection’); safety and a supportive environment (‘Safety’); learning, competence, education, skills and employability (‘Learning’); and agency and resilience (‘Agency’)] and 27 sub-domains of adolescent well-being, or as general statements of well-being. About 1 509 904 responses (>99.9%) were classified. Since each response could contain multiple ‘wants’, 1 714 027 individual ‘wants’ were identified at the domain level and 2 072 657 at the sub-domain level. Respondents were from 89 countries, with >99.9% from low- or middle-income countries (LMICs) and 1 397 735 (92.5%) from three WHO Regions (Africa (AFR); 48.9%), South-East Asia (SEAR); 33.3%; and Eastern Mediterranean (EMR); 10.3%, 112 125 (7.4%) from the Americas (AMR) but only 138 (<0.1%) and *N* = 6 (<0.1%) from the European (EUR) and Western Pacific Regions (WPR), respectively. About 90.4% were youth (15–24 years), and 50.5% self-identified as female. The greatest number of ‘wants’ related to the ‘Learning’ domain (36.3%), followed by the ‘Safety’ (28.4%) and ‘Health’ (20.9%) domains. Despite under-representation of high-income countries, AMR, and especially EUR and WPR, the results showed that young people gave importance to all five domains of well-being, especially ‘Learning’; ‘Safety’; and ‘Health’, across all four WHO Regions with adequate representation. The results emphasize that adolescent well-being is multi-faceted and that multi-sectoral policies and programmes will be essential to the successful promotion of adolescents’ health and well-being.

Key messages

- This massive online survey evaluated the priorities, concerns and aspirations of over 1.5 million young people by asking them to complete the sentence: ‘To improve my well-being, I want…’
- ‘Wants’ related to three domains (learning, competence, education, skills and employability; safety and a supportive environment; and good health and optimum nutrition) were suggested most often across all four World Health Organization (WHO) Regions with adequate representation (Africa, South East Asia, Eastern Mediterranean, Americas), though the good health and optimum nutrition domain was particularly emphasized in the Eastern Mediterranean Region and the learning, competence, education, skills and employability domain particularly emphasized in the other three Regions.
- ‘Wants’ related to all five domains of adolescent well-being were suggested in all the four WHO Regions with adequate representation, which underscores its multi-component nature and the importance of multi-sectoral policies and programmes to achieve ‘what young people want’ to promote their well-being.
- Young people’s ‘wants’ should be taken into account by policies and programmes that aim to support their health and well-being and this survey provides information to contribute to that.

## Introduction

Young people (10–24 year-olds) are increasingly seen as being entitled to have a say in issues that affect their lives (UNICEF 1989, Clark *et al.* 2020). Also, knowing what young people want is essential to designing and implementing effective policies and programmes to promote their health and well-being (OECD 2017, PMNCH 2020). Co-designing specific policies or programmes with young people has been undertaken increasingly in recent years (Vella-Brodrick *et al.* 2017) including using person-centred design (Doyle *et al.* 2019). Crowdsourcing of information from young people has sometimes been employed to elicit ideas related to their well-being or programme design (e.g. Hlatshwako *et al.* 2025).

Since 2015, with support from Fondation Botnar and others, the Partnership for Maternal, Newborn and Child Health (PMNCH), and the World Health Organization (WHO) have been working with partners within the United Nations H6+ Technical Working Group on Adolescent Health and Well-being and other stakeholders on the Adolescent Well-being Initiative. Among other things, in consultation with young people, this initiative has developed a UN H6 + definition (Adolescents thrive and are able to achieve their full potential) and conceptual framework for adolescent well-being (Ross *et al.* 2020). The framework identified five domains of adolescent well-being: good health and optimum nutrition; connectedness, positive values, and contribution to society; safety and a supportive environment; learning, competence, education, skills, and employability; agency and resilience. These domains were further divided into a total of 27 sub-domains (Fig. 1) (Ross *et al.* 2020). This definition and conceptual framework drew on findings from regional consultations with young people in all six WHO Regions. To further extend the consultation with young people, in line with PMNCH’s commitment to meaningful adolescent and youth engagement (PMNCH 2020), in the build-up to the Global Forum for Adolescents that was held in 2023 (Ross *et al.* 2023), the PMNCH Secretariat organized the multi-country What Young People Want (WYPW) online survey with a particular focus on young people living in low- and middle-income countries (LMICs). Young people were invited to complete the statement: ‘To improve my well-being, I want…’ (https://wypw0.1point8b.org/en).

Extensive youth consultation processes in high-income countries—such as the European Union's structured 18-month Youth Dialogue cycles (European Youth Forum n.d.), Australia's annual survey of over 17 400 youth focusing on stress management and body image concerns (Mission Australia 2024), and Canada's comprehensive policy consultations emphasizing meaningful work and civic engagement (Government of Canada 2022, Students Commission of Canada n.d.)—predominantly capture perspectives from well-resourced settings with established social safety nets. Similarly, previous studies such as those by Baillergeau *et al.* (2015) and Deutsch and Theodorou (2010) have provided valuable insights into youth aspirations, particularly within high-income contexts. Baillergeau *et al.* (2015) explored how young Europeans formulate desirable futures and found that they commonly emphasized the role of education, consumption, and civic engagement in shaping aspirations. However, their study relied on structured formats and was geographically limited to European youth. Also, Deutsch and Theodorou (2010) examined adolescent perceptions of social mobility and belonging in Cyprus, but their work was based on a small sample and did not incorporate participatory or open-text methods.

In contrast, the WYPW survey aimed to crowdsource the aspirations of a very large number of young people (10–24 years) related to their well-being and to focus particularly on young people living in low- and middle-income countries (LMICs). Also, the aim was to accommodate varying literacy levels by using a single, simple prompt—‘To improve my well-being, I want…’ and to allow individuals to express themselves in their own words rather than being constrained to select predefined options. A further methodological aim was to use natural language processing (NLP) with thematic analysis to classify the open-ended responses in order to offer a rich, bottom-up understanding of what young people want for their well-being. This design not only aimed to amplify youth voices from traditionally marginalized regions but also to advance methodological innovation in participatory global health research.

Here, we report the main results from this survey.

A more detailed report of the survey’s findings is available at https://www.1point8b.org/_files/ugd/c1e164_ce071750edd34f4d91c9153e53440ef1.pdf?index=true and an interactive dashboard of the survey’s preliminary results prior to data cleaning is available at https://wypw.1point8b.org/en. The dashboard allows users to search by country and/or domain of adolescent well-being and to see a sample of up to 1000 responses within each search.

## Methods

The WYPW survey aimed to capture open-ended responses from young people to a single prompt: ‘To improve my well-being, I want…’ This format aimed to accommodate young people with varying literacy levels and time constraints while allowing culturally-specific expressions of their ‘wants’. The WYPW survey also particularly targeted young people from low- and middle-income countries (LMICs).

### Data collection

PMNCH promoted the survey on digital channels and invited attendees at events organized by PMNCH or partners to participate. A Quick Response (QR) code was provided so that individuals could use their smartphones to participate. The code directed them to a WhatsApp-based chatbot where they could respond to the survey directly. The survey was anonymous and no personal identifiers were requested. Complying with the European Union’s General Data Protection Regulation (GDPR) (European Union 2016), if a respondent was under 16 years of age, digital means of data collection were not used by the adolescent respondent and permission from a parent or legal guardian was obtained, in addition to being open to any young people online, PMNCH partner organizations in 16 low- and middle-income countries (LMICs) (Brazil, Cameroon, Colombia, Egypt, Ghana, India, Indonesia, Kenya, Liberia, Malawi, Nigeria, Sierra Leone, Tanzania, Uganda, Zambia and Zimbabwe) also trained community mobilizers who organized mass collection efforts in schools, places of worship, at sports events, community hearings, national events and through door-to-door campaigns. At these events, respondents who had a smartphone could enter their response directly, but others could write their response and basic demographic details (age in years, gender and country of residence) on a piece of paper and give it to a community mobilizer who entered the data on to the WhatsApp chatbot on their behalf. To maximize access to the survey, respondents were given the option to submit their responses in any language.

### Data management

Prior to analysis, responses were cleaned to ensure quality. This involved removing duplicate entries and data from respondents who did not meet the minimum and maximum age or consent requirements; excluding entries where either the respondent’s age or country were missing; correcting obvious input errors; standardizing response text such as putting words into a uniform case, removal of unnecessary punctuation and translating non-English responses using Microsoft Azure Translation services, integrated within the broader Microsoft Azure cloud infrastructure.

### Data analysis

The analysis of the demographic data was performed using descriptive statistics, while the textual responses to the question about ‘wants’ were examined through deductive thematic analysis (Braun and Clarke 2022) to identify themes (domains) and subthemes (subdomains) in an iterative process. First, text responses were tokenized (split into words) using standard natural language processing (NLP)-based word matching techniques (Michelbacher 2013, Wiedemann and Fedtke 2022). Stop words such as ‘a’, ‘the’, ‘is’, ‘and’ were removed, and stemming/lemmatization was applied to reduce words to their root form.

Word count statistics were calculated to identify the most frequently used words and phrases in the responses. Frequency distributions and word clouds were used as visual tools to help identify key themes.

An initial manual check of 1% of the responses was used to develop a codebook. This showed that there were some responses that were very non-specific such as ‘nice’, ‘express myself’, ‘sleep better’, and ‘time for me’. These were assigned to a code for ‘broad well-being’. All the other responses could be assigned to one or more of the 27 subdomains of the adolescent well-being framework (Ross *et al.* 2020), with the exception of 100 responses, which were so non-specific as to be meaningless, such as ‘whatever’, ‘I didn’t think’, ‘squid out’. Frequency analyses were also performed within each domain and subdomain to uncover distinctive language patterns per domain and subdomain.

Using IBM SPSS Statistics software version 30.0.0, a coding scheme was developed based on the adolescent well-being domains and subdomains. Manual coding was repeated with another 4% subset of the responses to balance the efficiency and accuracy of the coding and then automated using syntax routines for the full dataset. Each response was analysed for the presence or absence of specific keywords and phrases corresponding to each domain and subdomain. For example, if a respondent mentioned ‘better physical and mental health’, this response was categorized under the domain of ‘good health and optimum nutrition’, was further coded under the subdomains of ‘physical health and capacities’ and ‘mental health and capacities’.

## Results

### Respondents

The survey ran from October 2022 to October 2023 and elicited 1 510 004 responses from young people aged 10–24 years. Table 1 presents the number of respondents by WHO Region, age group and gender. This shows that the vast majority (90.4%) were youth (15–24 years), with only 9.6% young adolescents (10–14 years). Slightly more respondents self-identified as women (50.5%) than as men (46.2%), with a total of 41 009 (2.7%) identifying as other genders, including agender, genderfluid, transman, transwoman, two-spirit, non-binary, and other. Only 8621 (0.6%) either preferred not to disclose their gender (*n* = 8575) or left this question blank (*n* = 46).

**Table 1:** Number of respondents by WHO region, age group, and gender.

WHO region	Man				Woman				Other or prefer not to disclose				Total			
	10–14 years *n* (row %)	15–19 years *n* (row %)	20–24 years *n* (row %)	Total *n* (row %)	10–14 years *n* (row %)	15–19 years *n* (row %)	20–24 years *n* (row %)	Total *n* (row %)	10–14 years *n* (row %)	15–19 years *n* (row %)	20–24 years *n* (row %)	Total *n* (row %)	10–14 years *n* (row %)	15–19 years *n* (row %)	20–24 years *n* (row %)	Total *n* (column %)
**Africa**	45 441 (12.8%)	185 469 (52.3%)	123 528 (34.9%)	354 438 (48.0%)	52 628 (13.8%)	207 713 (54.5%)	120 629 (31.7%)	380 970 (51.6%)	256 (8.6%)	1746 (59.0%)	959 (32.4%)	2961 (0.4%)	98 325 (13.3%)	394 928 (53.5%)	245 116 (33.2%)	738 369 (48.9%)
**Eastern Mediterranean**	0 (0.0%)	34 624 (44.5%)	43 175 (55.5%)	77 799 (49.8%)	0 (0.0%)	35 312 (45.1%)	43 029 (54.9%)	78 341 (50.2%)	0 (0.0%)	23 (50.0%)	23 (50.0%)	46 (<0.1%)	0 (0.0%)	69 959 (44.8%)	86 227 (55.2%)	156 186 (10.3%)
**Europe**	0 (0.0%)	11 (33.3%)	22 (66.7%)	33 (23.9%)	4 (4.6%)	28 (32.2%)	55 (63.2%)	87 (63.0%)	0 (0.0%)	12 (66.7%)	6 (33.3%)	18 (13.0%)	4 (2.9%)	51 (37.0%)	83 (60.1%)	138 (<0.1%)
**Americas**	11 805 (25.5%)	19 861 (42.9%)	14 680 (31.7%)	46 346 (41.3%)	12 830 (22.2%)	24 346 (42.1%)	20 608 (35.7%)	57 784 (51.5%)	439 (5.5%)	3879 (48.5%)	3677 (46.0%)	7995 (7.1%)	25 074 (22.4%)	48 086 (42.9%)	38 965 (34.8%)	112 125 (7.4%)
**South-East Asia**	7127 (3.2%)	100 783 (45.9%)	111 456 (50.8%)	219 366 (43.6%)	14 836 (6.1%)	122 448 (49.9%)	107 922 (44.0%)	245 206 (48.7%)	3 (<0.1%)	13 683 (35.4%)	24 922 (64.6%)	38 608 (7.7%)	21 966 (4.4%)	236 914 (47.1%)	244 300 (48.6%)	503 180 (33.3%)
**Western Pacific**	0 (0.0%)	0 (0.0%)	2 (100.0%)	2 (33.3%)	0 (0.0%)	1 (50.0%)	1 (50.0%)	2 (33.3%)	0 (0.0%)	0 (0.0%)	2 (100.0%)	2 (33.3%)	0 (0.0%)	1 (16.7%)	5 (83.3%)	6 (<0.1%)
**Total**	64 373 (9.2%)	340 748 (48.8%)	292 863 (42.0%)	697 984 (46.2%)	80 298 (10.5%)	389 848 (51.1%)	292 244 (38.3%)	762 390 (50.5%)	698 (1.4%)	19 343 (39.0%)	29 589 (59.6%)	49 630 (3.3%)	145 369 (9.6%)	749 939 (49.7%)	614 696 (40.7%)	1 510 004 (100.0%)

This table presents the distribution of survey respondents by age and gender across the six WHO regions (Africa, Eastern Mediterranean, Europe, the Americas, South-East Asia, and Western Pacific). It includes the number and percentage of individuals in three age groups—10–14, 15–19, and 20–24 years—categorized by gender: Man, Woman, and Other or Prefer not to disclose. The table provides total counts and percentages for each category and region, along with overall totals. The great majority (90.8%) self-identified as either being a man or a woman. Almost half (49.7%) were aged 15–19 years and 40.7% were aged 20–24 years, with just under 10% (9.6%) aged 10–14 years. At least 100,000 respondents came from four WHO regions (Africa, Eastern Mediterranean, the Americas, and South-East Asia, with nearly half of all respondents from the African region and one third from South East Asia, while less than 0.1% came from Europe or from the Western Pacific. WHO = World Health Organization; n = number of respondents; y = age in years.

Over three-quarters of the respondents (1 146 896; 76.0%) came from lower middle-income countries (lower MICs); 251 163 (16.6%) from low-income countries (LICs), 111 900 (7.4%) from upper middle-income countries (upper MICs), and only 43 (<0.1%) from high-income countries (HICs). About 1 496 277 respondents (97.9%) came from 15 countries: India (28.5%), Egypt (10.3%), Nigeria (9.3%), Uganda (7.5%), Tanzania (5.5%), Colombia (5.3%), Indonesia (4.8%), Zambia (4.0%), Liberia (3.6%), Cameroon (3.6%), Zimbabwe (3.6%), Malawi (3.4%), Ghana (3.3%), Kenya (3.0%), and Sierra Leone (2.1%). All 15 were among the countries where in-person data collection efforts had been organized by PMNCH grantees and other civil society partners.

The vast majority of the 1 509 860 (99.9%) originated from four WHO regions. Nearly half, 48.9% (*n* = 738 369), were from AFR followed by 33.3% (*n* = 503 180) from SEAR while EMR accounted for 10.3% (*n* = 156 186). AMR accounted for 7.4% (*n* = 112 125). In contrast, very few respondents (*n* = 138) came from EUR or from the WPR (*n* = 6) (Fig. 1).

**Figure 1:**
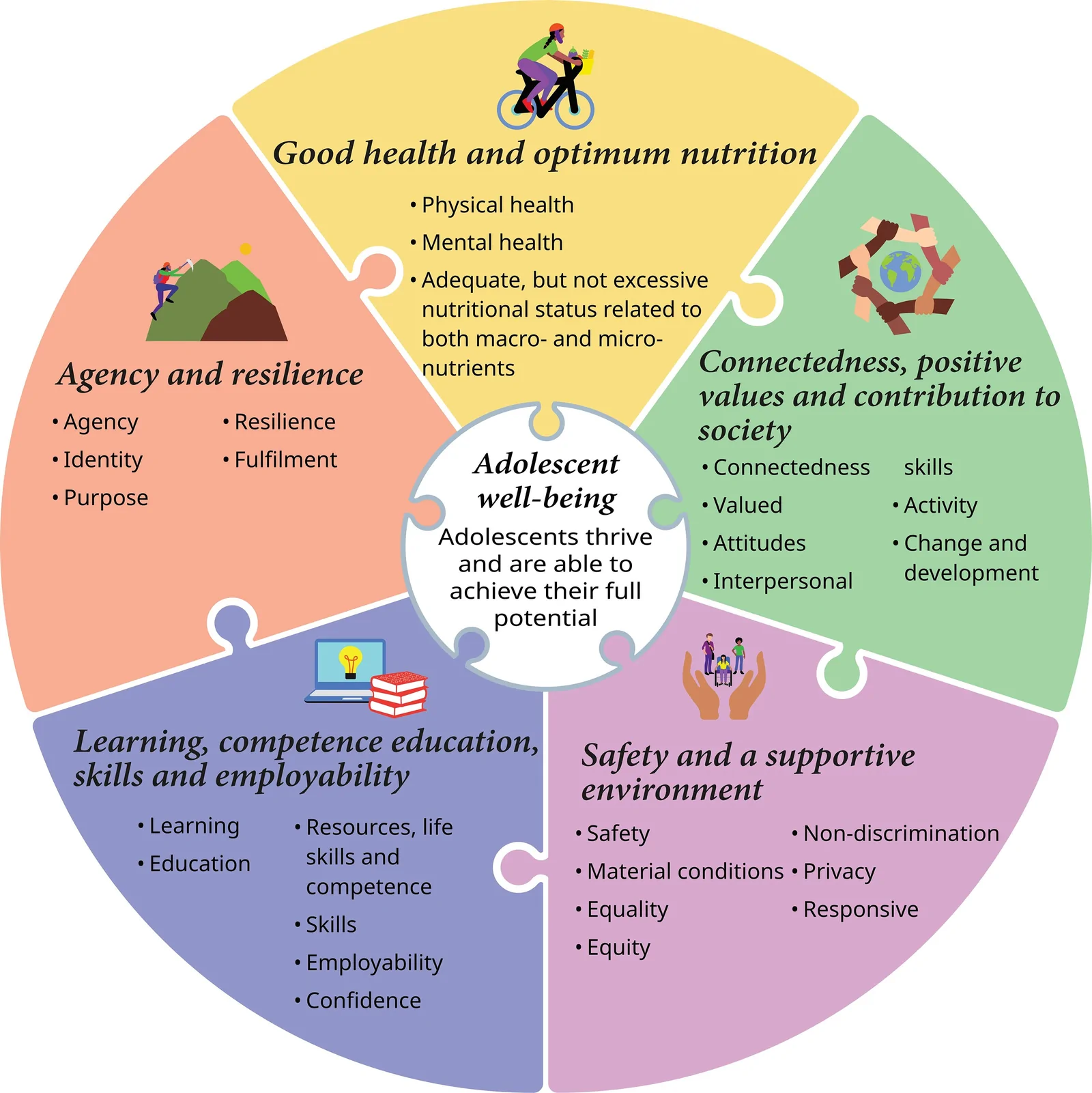
Domains and sub-domains of adolescent well-being. This figure visually maps the five core domains of adolescent well-being: (1) Good Health and Optimum Nutrition, (2) Connectedness, Positive Values and Contribution to Society, (3) Safety and a Supportive Environment, (4) Learning, Competence, Education, Skills and Employability, and (5) Agency and Resilience. Each domain branches into multiple sub-domains, such as physical and mental health, interpersonal skills, equity, education, employability, and identity. The layout as a jigsaw illustrates how these elements are interconnected collectively shaping holistic adolescent well-being and emphasizing the multidimensional nature of well-being. *Source of data:* Ross *et al.* 2020.

### Wants

Since a single response could sometimes relate to more than one domain of adolescent well-being, the 1 510 004 responses included 1 714 027 ‘wants’ that could be coded to one or more domains of adolescent well-being or to ‘general well-being’. An example of how one response could refer to multiple domains was: ‘To improve my well-being I want free sanitary pads in homes’. It refers to a material condition, which is a subdomain within the ‘safety and a supportive environment’ domain, as well as the ‘physical health and capacities’ subdomain within the ‘good health and optimum nutrition domain’.

An overview of the most frequent phrases within the 1 510 004 responses is shown in the word cloud in Fig. 2. The most frequent phrases related either to the ‘learning, competence, education, skills, and employability domain’—for example, ‘good education’, ‘education and learning opportunity’, ‘learning opportunity’, ‘quality education’, ‘education and economic opportunity’, ‘job opportunity’, and ‘government job’—or to the ‘good health and optimum nutrition domain’—for example ‘mental health’, ‘good health’, ‘reproductive health’ and ‘sexual and reproductive health’. Some specific examples of responses are given in Table 2.

**Figure 2:**
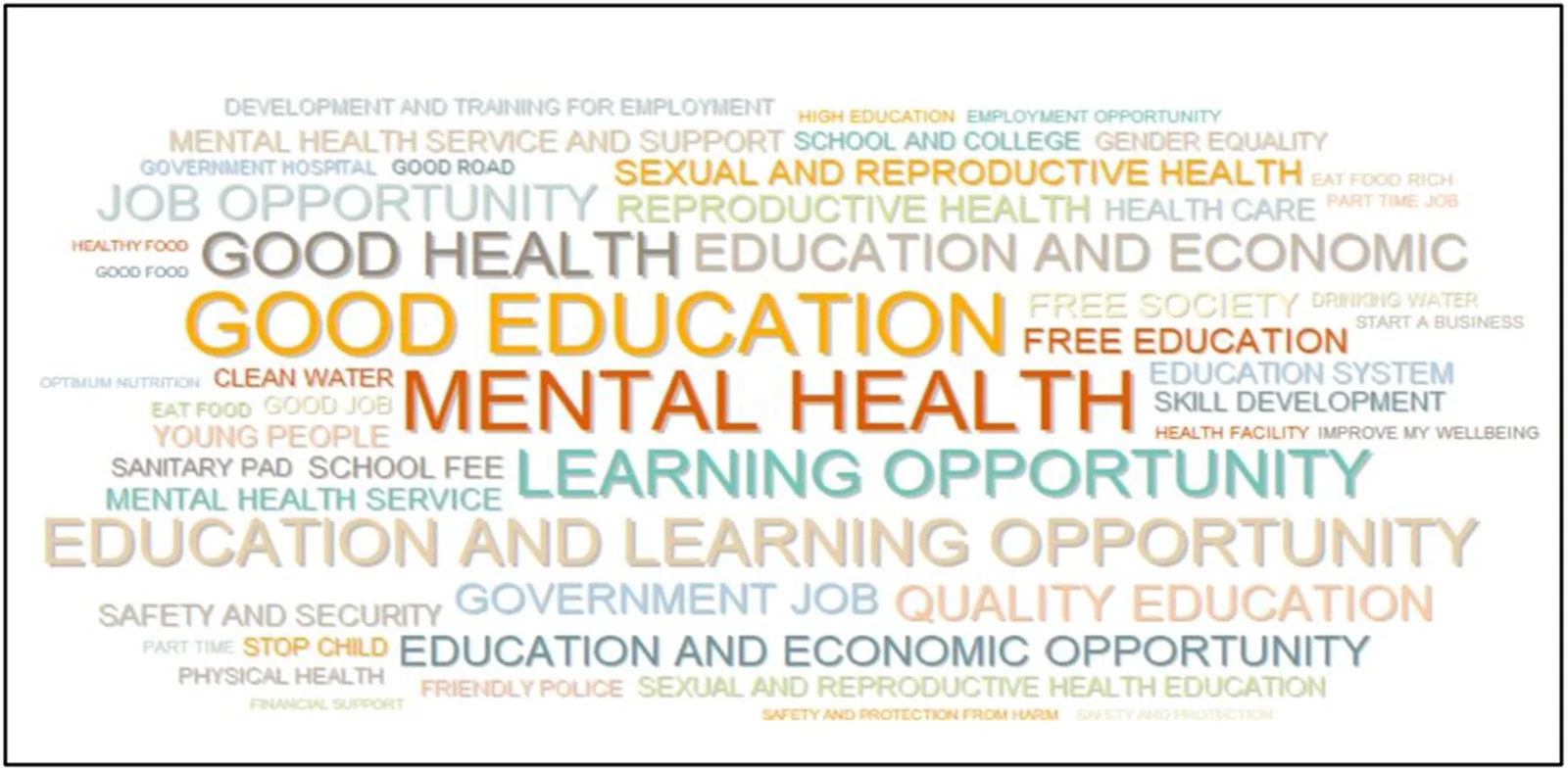
Word Cloud (Phrases) from Participants' Responses. This word cloud is composed of phrases extracted from adolescent participants' responses, reflecting their priorities and aspirations. The size of each phrase indicates its relative frequency, highlighting diverse concerns across all five domains of adolescent well-being. Prominent phrases include ‘GOOD EDUCATION’, ‘LEARNING OPPORTUNITY’, EDUCATION AND LEARNING OPPORTUNITY', and ‘QUALITY EDUCATION’; ‘MENTAL HEALTH’; ‘GOVERNMENT JOB’, ‘JOB OPPORTUNITY’ and ‘EDUCATION AND ECONOMIC OPPORTUNITY’; ‘SEXUAL AND REPRODUCTIVE HEALTH’ and ‘REPRODUCTIVE HEALTH’.

**Table 2:** Examples of responses to the question ‘“To improve adolescents” well-being, I want….’.

Domains	Examples
**Good health and optimum nutrition**	‘…..a quality health service’ ‘….to cultivate healthy sleeping, eating, and exercise habits to increase my physical and mental safety’ ‘…..mental health support’
**Connectedness, positive values, and contribution to society**	‘…to be valued and loved by my family’ ‘….more empowerment for young people to be included in decision making’ ‘….to become an advocate for change’
**Safety and a supportive environment**	‘….a safe place in my country’ ‘….better housing conditions’ ‘….gender equality’
**Learning, competence, education, skills, and employability**	‘….to get a good job after I leave school’ ‘….to acquire business management skills’ ‘….to stay in school and finish school’
**Agency and resilience**	‘…..all members to be empowered to participate in their community and democracy’ ‘…Improved self-esteem and confidence’ ‘….be able to overcome my difficulties’

This table provides examples of adolescent responses on what they want to improve their well-being, organized into the five domains of adolescent well-being. Under Good health and optimum nutrition, responses included wanting a quality health service, promotion of healthy lifestyles and mental health support. Connectedness, positive values, and contribution to society included responses related to family support, youth empowerment, and civic engagement. Safety and a supportive environment included wanting a safe place, better housing conditions and gender equality. Learning, competence, education, skills, and employability included “wants” related to future employment, skills related to business management and aspirations for education. Finally, Agency and resilience included responses related to universal empowerment and democracy, improved self-esteem and confidence, and the ability to overcome adversity.

The manual analysis conducted by two independent reviewers (R.D.J, D.A.R.) of 100 random responses, who independently coded each response into one or more subdomains of adolescent well-being or as broad well-being or as being uncodable, showed that they fully agreed with 49 of the computer-coded responses for both the domain(s) and subdomain(s), partially agreed with 20, and disagreed with 31.

Only 2.4% of ‘wants’ were coded to broad well-being (range 1.2–3.6% by Region).

### ‘Wants’ by domain of adolescent well-being

Overall, across the whole set of respondents there were at least 40 000 ‘wants’ within each of the five domains of adolescent well-being, but the number of ‘wants’ varied considerably by domain (Table 3 and Fig. 3). The domain with the most ‘wants’ was ‘learning, competence, education, skills, and employability’ (*n* = 623 357), followed by ‘safety and a supportive environment’ (*n* = 485 977), ‘good health and optimum nutrition’ (*n* = 358 282), ‘connectedness, positive values and contribution to society’ (*n* = 160 591), and ‘agency and resilience’ (*n* = 44 983). This ranking of ‘wants’ was the same in the responses from all three age groups (10–14 years, 15–19 years, 20–24 years) (Table 3).

**Figure 3:**
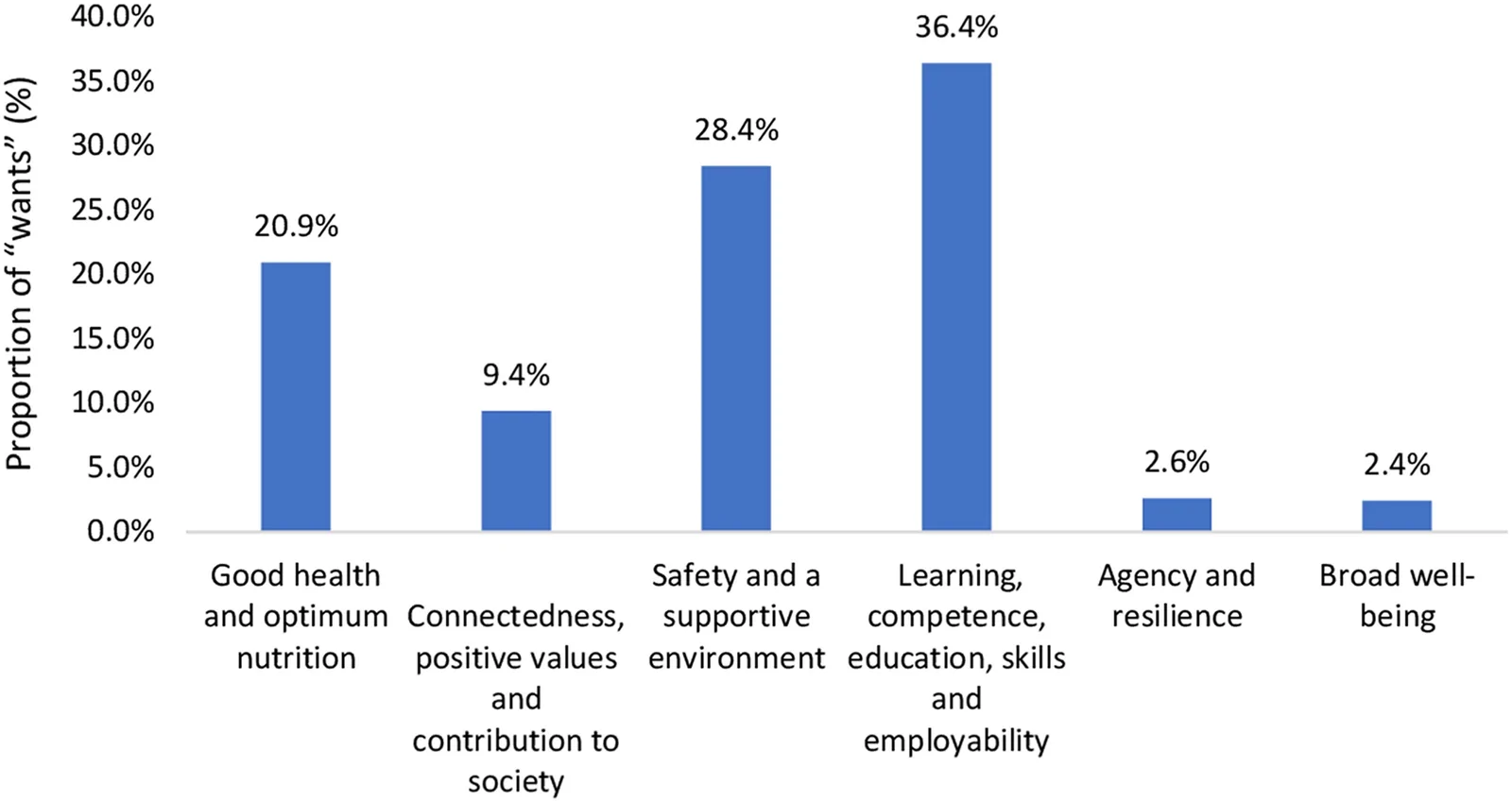
Young people's ‘wants' by adolescent well-being domain. This bar chart illustrates the distribution of young people's expressed ‘wants' across the five domains of adolescent well-being. The chart shows the percentage of responses per domain.: (1) Good Health and Optimum Nutrition (20.9%), (2) Connectedness, Positive Values and Contribution to Society (9.4%), (3) Safety and a Supportive Environment (28.4%), (4) Learning, Competence, Education, Skills and Employability (36.4%), and (5) Agency and Resilience (2.6%). In addition 2.4% of the responses were classified as “Broad well-being”.

**Table 3:** Participants’ ‘wants’ by domain^a^, subdomain^a^, and age group^a,b^.

Domains and subdomains of adolescent well-being	Total			
	10–14 years *n* (column %)	15–19 years *n* (column %)	20–24 years *n* (column %)	Total *n* (column %)
**1. Good health and optimum nutrition**	29 719 (18.0%)	178 283 (21.0%)	150 280 (21.5%)	358 282 (20.9%)
**Physical health and capacities**	21 750 (10.7%)	117 731 (11.4%)	92 901 (11.1%)	232 382 (11.2%)
**Mental health and capacities**	11 498 (5.6%)	75 266 (7.3%)	65 147 (7.7%)	151 911 (7.3%)
**Optimal nutritional status and diet**	7475 (3.7%)	49 851 (4.8%)	42 949 (5.1%)	100 275 (4.8%)
**2. Connectedness, positive values and contribution to society**	12 585 (7.6%)	73 398 (8.6%)	74 608 (10.7%)	160 591 (9.4%)
**Connectedness**	1939 (1.0%)	9745 (0.9%)	9819 (1.2%)	21 503 (1.0%)
**Valued and respected**	1296 (0.6%)	5727 (0.6%)	6166 (0.7%)	13 189 (0.6%)
**Attitudes**	2610 (1.3%)	18 001 (1.8%)	14 544 (1.7%)	35 155 (1.7%)
**Interpersonal skills**	5765 (2.8%)	24 587 (2.4%)	24 693 (2.9%)	55 045 (2.7%)
**Activity**	1245 (0.6%)	4628 (0.5%)	4070 (0.5%)	9943 (0.5%)
**Change and development**	3682 (1.8%)	26 475 (2.6%)	30 928 (3.7%)	61 085 (2.9%)
**3. Safety and a supportive environment**	48 970 (29.7%)	234 652 (27.6%)	202 355 (29.0%)	485 977 (28.4%)
**Safety**	356 (0.2%)	8444 (0.8%)	12 889 (1.5%)	21 689 (1.0%)
**Material conditions**	500 (0.2%)	6540 (0.6%)	7349 (0.9%)	14 389 (0.7%)
**Equity**	36 876 (18.1%)	159 302 (15.5%)	121 969 14.5%)	318 147 (15.3%)
**Equality**	211 (0.1%)	5818 (0.6%)	9815 (1.2%)	15 844 (0.8%)
**Nondiscrimination**	425 (0.2%)	1607 (0.2%)	1462 (0.2%)	3494 (0.2%)
**Privacy**	3989 (2.0%)	30 786 (3.0%)	34 726 (4.1%)	69 501 (3.4%)
**Responsive**	9982 (4.9%)	41 080 (4.0%)	34 392 (4.1%)	85 454 (4.1%)
**4. Learning, competence, education, skills and employability**	68 040 (41.3%)	323 823 (38.1%)	231 494 (33.1%)	623 357 (36.4%)
**Learning**	1476 (0.7%)	8024 (0.8%)	9044 (1.1%)	18 544 (0.9%)
**Education**	54 829 (26.9%)	218 492 (21.2%)	112 450 (13.4%)	385 771 (18.6%)
**Resources, life skills and competencies**	14 519 (7.1%)	90 928 (8.8%)	98 430 (11.7%)	203 877 (9.8%)
**Skills**	11 350 (5.6%)	34 017 (3.3%)	21 882 (2.6%)	67 249 (3.2%)
**Employability**	1043 (0.5%)	11 978 (1.2%)	12 208 (1.5%)	25 229 (1.2%)
**Confidence that they can do things well**	4867 (2.4%)	34 097 (3.3%)	28 394 (3.4%)	67 358 (3.2%)
**5. Agency and resilience**	2705 (1.6%)	21 717 (2.6%)	20 561 (2.9%)	44 983 (2.6%)
**Agency**	138 (0.1%)	2665 (0.3%)	2542 (0.3%)	5345 (0.3%)
**Identity**	713 (0.4%)	6402 (0.6%)	5115 (0.6%)	12 230 (0.6%)
**Purpose**	714 (0.4%)	5234 (0.5%)	7601 (0.9%)	13 549 (0.7%)
**Resilience**	1386 (0.7%)	9608 (0.9%)	6859 (0.8%)	17 853 (0.9%)
**Fulfilment**	129 (0.1%)	2702 (0.3%)	2978 (0.4%)	5809 (0.3%)
**Broad well-being**	2759 (1.7%)	18 685 (2.2%)	19 410 (2.8%)	40 854 (2.4%)
**Total no. of ‘wants’ by domain**	164 778 (9.6%)	850 558 (49.6%)	698 708 (40.8%)	1 714 044 (100.0%)

This table presents the distribution of adolescents' expressed ‘wants' across the five domains and 27 subdomains of well-being—categorized by age groups: 10–14, 15–19, and 20–24 years. Among 10 to 14 year olds, ‘wants' were predominantly centered around ‘Learning, competence, education, skills and employability', which accounted for 41.3% of responses. Additionally, ‘Safety and a supportive environment' was a significant ‘want', representing 29.7% of responses. Within the 15 to 19 year age group, ‘wants' related to ‘Learning, competence, education, skills and employability' remained a strong priority at 38.1%, but there was a small increase in responses related to ‘Good health and optimum nutrition' (21.0% compared to 18.0%), ‘Connectedness, positive values and contribution to society' (8.6% vs. 7.6%), and ‘Agency and resilience' (2.6% vs. 1.6%). Among 20 to 24 year olds, the proportion of responses related to ‘Learning, competence, education, skills and employability' remained the domain with the largest proportion of ‘wants' but this further declined to 33.1%. In contrast, responses regarding ‘Good health and optimum nutrition' rose slightly to 21.5%, driven by increases in the subdomains of ‘Mental health and competencies' (from 7.3% in the 15–19 age group to 7.7%) and ‘Optimal nutrition and diet' (from 4.8% to 5.1%).

^a^Where a response specified two ‘wants' from different subdomains within the same domain, these were classified as two ‘wants' in the analysis by subdomains but as one ‘want' for that domain as a whole. As a result, the total number of ‘wants' for the analysis by subdomains was 2,072,657 but was 1,714,044 for the analysis by domains.

^b^Limited to the four WHO regions with ≥100,000 respondents per region.

All the four Regions with at least 100 000 responses had the least wants from the ‘agency and resilience’ domain (range 1.3–3.4%) followed by ‘connectedness, positive values and contribution to society’ (range 7.2–11.8%; Table 4). All these four Regions had a similar proportion of wants that fell into the ‘safety and a supportive environment’ domain (range 25.9–30.0%) and this domain always ranked second. However, there were important differences across the Regions related to the ‘learning, competence, education, skills, and employability’ and the ‘good health and optimum nutrition’ domains where AFR, SEAR and AMR had a similar pattern with identical ranking with the ‘learning, competence, education, skills, and employability’ domain ranking first and ‘good health and optimum nutrition’ ranking third, whereas EMR had a very different pattern with ‘good health and optimum nutrition’ ranking first and ‘learning, competence, education, skills, and employability’ ranking third (Table 4).

**Table 4:** Participants’ ‘wants’ by domain^a^, subdomain^a^ and WHO Region^b^.

Domains and subdomains of adolescent well-being	Africa	Eastern mediterranean	Americas	South east asia	Total
	*n* (column %)	*n* (column %)	*n* (column %)	*n* (column %)	*n* (column %)
**1. Good health and optimum nutrition**	125 283 (16.2%)	91 744 (51.1%)	28 841 (20.9%)	112 358 (18.0%)	358 282 (20.9%)
**Physical health and capacities**	85 901 (9.3%)	52 343 (23.9%)	16 657 (9.7%)	77 444 (10.2%)	232 382 (11.2%)
**Mental health and capacities**	53 955 (5.8%)	31 252 (14.3%)	14 845 (8.7%)	51 823 (6.8%)	151 911 (7.3%)
**Optimal nutritional status and diet**	25 879 (2.8%)	34 421 (15.7%)	7656 (4.5%)	32 314 (4.3%)	100 275 (4.8%)
**2. Connectedness, positive values and contribution to society**	58 694 (7.6%)	12 868 (7.2%)	15 215 (11.0%)	73 791 (11.8%)	160 591 (9.4%)
**Connectedness**	8243 (0.9%)	7703 (3.5%)	2892 (1.7%)	2662 (0.4%)	21 503 (1.0%)
**Valued and respected**	4149 (0.4%)	227 (0.1%)	2572 (1.5%)	6235 (0.8%)	13 189 (0.6%)
**Attitudes**	14 428 (1.6%)	2104 (1.0%)	2119 (1.2%)	16 501 (2.2%)	35 155 (1.7%)
**Interpersonal skills**	21 025 (2.3%)	2386 (1.1%)	5565 (3.3%)	26 066 (3.4%)	55 045 (2.7%)
**Activity**	4668 (0.5%)	1016 (0.5%)	1607 (0.9%)	2646 (0.3%)	9943 (0.5%)
**Change and development**	21 828 (2.4%)	1512 (0.7%)	6033 (3.5%)	31 693 (4.2%)	61 085 (2.9%)
**3. Safety and a supportive environment**	231 611 (30.0%)	46 412 (25.9%)	38 537 (27.9%)	169 369 (27.1%)	485 977 (28.4%)
**Safety**	4577 (0.5%)	51 (0.0%)	1353 (0.8%)	15 701 (2.1%)	21 689 (1.0%)
**Material conditions**	4367 (0.5%)	301 (0.1%)	2293 (1.3%)	7425 (1.0%)	14 389 (0.7%)
**Equity**	179 651 (19.5%)	30 885 (14.1%)	20 019 (11.7%)	87 573 (11.5%)	318 147 (15.3%)
**Equality**	1221 (0.1%)	502 (0.2%)	580 (0.3%)	13 539 (1.8%)	15 844 (0.8%)
**Nondiscrimination**	1387 (0.2%)	40 (<0.1%)	293 (0.2%)	1774 (0.2%)	3494 (0.2%)
**Privacy**	24 428 (2.6%)	12 279 (5.6%)	8999 (5.3%)	23 776 (3.1%)	69 501 (3.4%)
**Responsive**	33 661 (3.6%)	5945 (2.7%)	11 192 (6.5%)	34 647 (4.6%)	85 454 (4.1%)
**4. Learning, competence, education, skills and employability**	330 324 (42.8%)	21 390 (11.9%)	46 909 (34.0%)	224 698 (36.0%)	623 357 (36.4%)
**Learning**	10 924 (1.2%)	492 (0.2%)	1760 (1.0%)	5367 (0.7%)	18 544 (0.9%)
**Education**	217 450 (23.6%)	4264 (1.9%)	29 282 (17.1%)	134 746 (17.7%)	385 771 (18.6%)
**Resources, life skills and competencies**	92 799 (10.1%)	2028 (0.9%)	12 056 (7.0%)	96 989 (12.8%)	203 877 (9.8%)
**Skills**	40 802 (4.4%)	7201 (3.3%)	5742 (3.4%)	13 500 (1.8%)	67 249 (3.2%)
**Employability**	10 317 (1.1%)	7594 (3.5%)	2397 (1.4%)	4919 (0.6%)	25 229 (1.2%)
**Confidence that they can do things well**	31 282 (3.4%)	5909 (2.7%)	5912 (3.5%)	24 250 (3.2%)	67 358 (3.2%)
**5. Agency and resilience**	16 795 (2.2%)	2307 (1.3%)	4546 (3.3%)	21 321 (3.4%)	44 983 (2.6%)
**Agency**	1205 (0.1%)	23 (<0.1%)	316 (0.2%)	3800 (0.5%)	5345 (0.3%)
**Identity**	5495 (0.6%)	1678 (0.8%)	1377 (0.8%)	3680 (0.5%)	12 230 (0.6%)
**Purpose**	4327 (0.5%)	171 (0.1%)	1401 (0.8%)	7642 (1.0%)	13 549 (0.7%)
**Resilience**	7412 (0.8%)	1697 (0.8%)	1925 (1.1%)	6815 (0.9%)	17 853 (0.9%)
**Fulfilment**	1387 (0.2%)	242 (0.1%)	346 (0.2%)	3833 (0.5%)	5809 (0.3%)
**Broad well-being**	9541 (1.2%)	4719 (2.6%)	4011 (2.9%)	22 573 (3.6%)	40 854 (2.4%)
**Total no. of ‘wants’ by domain**	772 248 (45.1%)	179 440 (10.5%)	138 059 (8.1%)	624 110 (36.4%)	1 714 044 (100.0%)

This table shows the participants' ‘wants' by domain, subdomain, and WHO Region for the four regions with at least 100000 responses (Africa, the Americas, the Eastern Mediterranean, and the South East Asian Regions). It highlights regional disparities in the proportions of ‘wants' related to the various aspects of well-being of young people across the five domains. The highest proportion of responses from young people living in three of the four WHO Regions related to ‘Learning, competence, education, skills, and employability' (Africa 42.8%, South East Asia 36.0%, the Americas 34.0%), In marked contrast, only 11.9% of responses from young people from the Eastern Mediterranean Region related to this domain, whereas 51.1% related to the ‘Good health and optimum nutrition' domain. About, one quarter to one-third of responses related to the ‘Safety and a supportive environment' domain in all four WHO Regions.

^a^Where a response specified two ‘wants' from different subdomains within the same domain, these were classified as two wants within the analysis by subdomains but as one ‘want' for that domain. As a result, the total number of ‘wants' for the analysis by subdomains was 2,072,657 but was 1,714,044 for the analysis by domains.

^b^Limited to the four WHO Regions with ≥100,000 respondents per Region.

Table 5 shows that the highest proportion of young people who self-identified as male and female mentioned ‘wants’ within the domain of ‘learning, competence, education, skills, and employability,’ while those identifying as agender, gender fluid, nonbinary, transwomen, transmen, and two-spirit—as well as those who selected ‘prefer not to say’—more often mentioned ‘safety and a supportive environment’. This group also mentioned ‘wants’ related to the ‘connectedness, positive values, and contribution to society’ domain more often than those who self-identified as male or female, with their responses often resulting in it ranking above education-related ‘wants’.

**Table 5:** Participants’ wants by domain^a^, subdomain^a^, and gender.

Domains and subdomains of adolescent well-being	Man	Woman	Other^b^ or preferred not to disclose^c^	Total
	*n* (column %)	*n* (column %)	*n* (column %)	*n* (column %)
**1. Good health and optimum nutrition**	165 772 (21.4%)	183 984 (21.0%)	8526 (13.7%)	358 282 (20.9%)
**Physical health and capacities**	108 586 (11.6%)	118 775 (11.2%)	5021 (6.7%)	232 382 (11.2%)
**Mental health and capacities**	68 739 (7.4%)	77 550 (7.3%)	5622 (7.5%)	151 911 (7.3%)
**Optimal nutritional status and diet**	46 633 (5.0%)	52 357 (4.9%)	1285 (1.7%)	100 275 (4.8%)
**2. Connectedness, positive values, and contribution to society**	70 449 (9.1%)	76 670 (8.7%)	13 472 (21.6%)	160 591 (9.4%)
**Connectedness**	10 115 (1.1%)	11 078 (1.0%)	310 (0.4%)	21 503 (1.0%)
**Valued and respected**	4413 (0.5%)	5623 (0.5%)	3153 (4.2%)	13 189 (0.6%)
**Attitudes**	17 189 (1.8%)	16 932 (1.6%)	1034 (1.4%)	35 155 (1.7%)
**Interpersonal skills**	23 362 (2.5%)	26 154 (2.5%)	5529 (7.4%)	55 045 (2.7%)
**Activity**	3676 (0.4%)	5421 (0.5%)	846 (1.1%)	9943 (0.5%)
**Change and development**	24 566 (2.6%)	29 161 (2.7%)	7358 (9.9%)	61 085 (2.9%)
**3. Safety and a supportive environment**	213 805 (27.6%)	253 476 (28.9%)	18 696 (29.9%)	485 977 (28.4%)
**Safety**	5498 (0.6%)	11 149 (1.0%)	5042 (6.8%)	21 689 (1.0%)
**Material conditions**	6142 (0.7%)	6891 (0.6%)	1356 (1.8%)	14 389 (0.7%)
**Equity**	145 901 (15.6%)	166 673 (15.6%)	5573 (7.5%)	318 147 (15.3%)
**Equality**	6036 (0.6%)	5971 (0.6%)	3837 (5.1%)	15 844 (0.8%)
**Nondiscrimination**	1560 (0.2%)	1887 (0.2%)	47 (0.1%)	3494 (0.2%)
**Privacy**	31 978 (3.4%)	34 989 (3.3%)	2534 (3.4%)	69 501 (3.4%)
**Responsive**	35 032 (3.8%)	48 222 (4.5%)	2200 (3.0%)	85 454 (4.1%)
**4. Learning, competence, education, skills, and employability**	287 989 (37.1%)	320 864 (36.6%)	14 504 (23.2%)	623 357 (36.4%)
**Learning**	7790 (0.8%)	10 040 (0.9%)	714 (1.0%)	18 544 (0.9%)
**Education**	172 591 (18.5%)	206 766 (19.4%)	6414 (8.6%)	385 771 (18.6%)
**Resources, life skills and competencies**	96 713 (10.4%)	100 717 (9.5%)	6447 (8.6%)	203 877 (9.8%)
**Skills**	31 322 (3.4%)	35 126 (3.3%)	801 (1.1%)	67 249 (3.2%)
**Employability**	12 826 (1.4%)	12 144 (1.1%)	259 (0.3%)	25 229 (1.2%)
**Confidence that they can do things well**	30 703 (3.3%)	34 989 (3.3%)	1666 (2.2%)	67 358 (3.2%)
**5. Agency and resilience**	17 232 (2.2%)	22 648 (2.6%)	5103 (8.2%)	44 983 (2.6%)
**Agency**	2018 (0.2%)	3065 (0.3%)	262 (0.4%)	5345 (0.3%)
**Identity**	5751 (0.6%)	6334 (0.6%)	145 (0.2%)	12 230 (0.6%)
**Purpose**	3320 (0.4%)	5802 (0.5%)	4427 (5.9%)	13 549 (0.7%)
**Resilience**	7903 (0.8%)	9653 (0.9%)	297 (0.4%)	17 853 (0.9%)
**Fulfilment**	2613 (0.3%)	2954 (0.3%)	242 (0.3%)	5809 (0.3%)
**Broad well-being**	20 011 (2.6%)	18 706 (2.1%)	2137 (3.4%)	40 854 (2.4%)
**Total no. of ‘wants’ by domain**	775 258 (45.2%)	876 348 (51.1%)	62 438 (3.6%)	1 714 044 (100.0%)

This table shows the participants' ‘wants' by domain, subdomain, and gender. It highlights both similarities and striking differences by gender that speak to deeper social dynamics and structural inequalities. Young people who self-identified as ‘man' and ‘woman' showed broadly similar distributions of ‘wants' with between one-fifth and over one third of their ‘wants' falling within the domains of ‘Learning, competence, education, skills and employability', ‘Safety and a supportive environment' and ‘Good health and optimum nutrition'. However, those who identified as being agender, genderfluid, transman, transwoman, two-spirit, non-binary or other or who preferred not to disclose their gender exhibited a distinct pattern of ‘wants' with 21.6% of ‘wants' related to ‘Connectedness, positive values and contribution to society' relative to 2.2% and 2.6% for ‘men' and ‘women', respectively. Similarly, a much higher proportion reported wants related to ‘Agency and resilience' (8.2% vs.2.2% and 2.6% for ‘men' and ‘women', respectively.

^a^Where a response specified two ‘wants' from different subdomains within the same domain, these were classified as two wants within the analysis by subdomains but as one ‘want' for that domain. As a result, the total number of ‘wants' for the analysis by subdomains was 2,072,657 but was 1,714,044 for the analysis by domains.

^b^Includes those who self-identified as agender, genderfluid, transman, transwoman, two-spirit, non-binary, and other.

^c^Including the 8575 who preferred not to disclose their gender and the 46 who left this question blank.

Although the absolute proportions of ‘wants’ differed by country income group, the ranking was always the same as in the overall distribution of «wants» (data not shown).

### ‘Wants’ by sub-domain of adolescent well-being

Some responses included ‘wants’ from two or more sub-domains within the same domain. As a result there were 2 072 657 ‘wants’ related to the 27 sub-domains. An example of a response that spanned multiple subdomains within a single domain was ‘To improve my well-being I want to reduce education fees’. This refers to the domain of ‘learning, competence, education, skills, and employability’ (1 response for domains) but was allocated to the subdomains of ‘resource’ and ‘education’ (2 ‘wants’ for subdomains).

Overall, the most represented sub-domains were ‘education’ (18.6%) within the ‘learning, competence, education, skills, and employability’ domain, followed by ‘material conditions’ (15.3%) within the ‘safety and a supportive environment’ domain, and ‘physical health and capacities’ (11.2%) within the ‘good health and optimum nutrition’ domain (Table 3).

As might be expected, the proportion of ‘wants’ related to ‘employability’ increased with age (Table 3). Other subdomains that were mentioned more by older young people included ‘a responsive environment’, ‘mental health and capacities’, ‘being valued and respected’, ‘nondiscrimination’, ‘equality’, and ‘equity’.

There were important differences between the four WHO Regions with at least 100 000 responses in the ‘wants’ by sub-domain (Table 4). While young people from AFR and SEAR both mentioned ‘wants’ from the ‘education’ sub-domain most frequently (23.6% and 17.1%, respectively), the second most frequently mentioned sub-domain in AFR was ‘material conditions’ (19.5%) but was ‘employability’ (12.8%) in SEAR. In contrast, young people in EMR mentioned ‘wants’ related to the subdomains of ‘education’ (3.3%) and ‘employability’ (0.9%) much less frequently than their peers from the other three WHO Regions. On the other hand, they frequently mentioned ‘wants’ related to all the three subdomains in the ‘good health and optimum nutrition’ domain: ‘physical health and capacities’ (23.9%), ‘mental health and capacities’ (14.3%), and ‘optimal nutritional status and diet’ (15.7%).

The proportion of ‘wants’ mentioned by respondents who identified as men and women were relatively similar (Table 4). However, young people who identified their gender as anything but man or woman mentioned wants that fell within the subdomains ‘valued and respected’, ‘equality’, ‘identity’, ‘connectedness’, and ‘non-discrimination’ more often (Table 5).

As for the analysis by domains, there were relatively few differences in the sub-domains of the ‘wants’ by country income group (data not shown). ‘Wants’ related to ‘education’ and ‘material conditions’ ranked one and two across all three country income groups (upper MICs, lower MICs, and LICs). However, respondents from LICs and lower MICs particularly mentioned ‘wants’ related to fundamental needs such as ‘education’, ‘material conditions’, ‘employability’, and ‘physical health and capacities’ while respondents from upper MICs tended to mention ‘wants’ related to ‘mental health and capacities’, ‘safety’, ‘connectedness’ and being ‘valued and respected’ more than young people from LICs and lower MICs.

## Discussion

It is essential that policy makers and programme managers know young people’s own views on what they consider is most important to their well-being and take these into account as they design, implement and evaluate programmes that aim to promote young people’s well-being. As far as we are aware, with over 1.5 million responses, the What Young People Want survey is the largest survey ever undertaken to establish this. As such, it demonstrates the potential of multi-country crowdsourcing.

Although the respondents were self-selected, special measures were taken in 16 LMICs to ensure that a substantial, diverse number of young people were reached. These measures included contacting young people at in-person events and facilitating submission of their responses to ensure that the views of respondents who did not have access to the internet were captured. A very high proportion of the responses came through these in-person events as indicated by the fact that all 15 of the countries from which the greatest number of responses came were where in-person data collection efforts had been organized by PMNCH grantees and other civil society partners. All of the 16 countries where in-person data collection efforts took place were LMICs in AFR, AMR, EMR and SEAR. However, the results cannot be considered to be globally representative, with particular under-representation of young people from the European and Western Pacific Regions, from high-income countries and with 97.9% of responses coming from 15 LMICs. This limitation affects the generalizability of our findings. Specifically, the survey tells us virtually nothing about the ‘wants’ of young people living in EUR or WPR or in high-income countries. To address this gap, we propose that any future survey that wishes to be globally representative should ensure that there is in-person mobilization of responses from countries in all six WHO Regions and that digital mobilization is done in all the major languages of the world.

The survey is particularly unusual in mainly presenting the views of young people from LMICs, as most surveys in the literature have focused on young people from HICs (e.g. Deutsch and Theodorou 2010, Baillergeau *et al.* 2015). Although young adolescents, who needed parental consent to participate, were under-represented with 9.6% of responses, there were still over 145 000 responses from 10- to 14-year olds. There was a roughly equal representation of responses from young people who self-identified as either a woman (*n* = 762 390) or as a man (*n* = 697 984), and substantial representation of those who self-identified as other genders (*n* = 41 009) or either chose not to disclose their gender or left the question blank (*n* = 8621).

Key factors in the successful uptake of the survey were that it only included one simple question (‘To improve my well-being, I want…’), which could be answered in any language, employing a Chatbot on the widely-used WhatsApp platform for data capture and the active promotion of the survey both at the global level and within 16 LMICs, where most of the mobilization of responses to the survey was done by youth-led organizations.

The analysis of self-reported, open-text data in this study presented several challenges, particularly regarding interpretation variability stemming from the brevity, ambiguity, and context-dependence of responses. To mitigate these limitations, we developed clear coding guidelines and utilized qualitative data analysis software to efficiently manage and identify patterns within the data. Automation based on computer algorithms designed to match words and phrases that were commonly associated with each of the domains and subdomains of the adolescent well-being framework in an initial manual coding of 1% of the responses, the subsequent independent check of a randomly-selected 100 responses showed that this automated coding had limitations, with 69% of the manually coded responses showing complete or partial agreement with the computer-based coding. The 31% disagreement rate suggests that while broad thematic patterns are likely to be robust given the survey’s large sample size, they may have implications for result interpretation. First, misclassification may not be randomly distributed across domains, potentially leading to systematic over- or under-representation of certain themes. For instance, responses related to mental health may have been more susceptible to misclassification due to varied cultural expressions of psychological well-being. At the same time, concrete ‘wants’ like ‘clean water’ may have been more reliably classified.

While we recognize the critical importance of capturing nuances such as idiomatic expressions, cultural references, and non-standard dialects that can significantly impact coding accuracy, it is essential to note that back-translations or checks by native speakers were not feasible due to the scale of the exercise, which involved over 1.5 million responses, and the lack of access to native speakers for certain languages. Although we implemented natural language processing and manual validation, these measures alone may not fully encapsulate the complexities of language and context.

The ‘wants’ of the respondents revealed important patterns. ‘Wants’ related to the domains of ‘learning, competence, education, skills and employability’; ‘safety and a supportive environment’; and ‘good health and optimum nutrition’ were suggested most often across all four World Health Organization (WHO) Regions with adequate representation, though the ‘good health and optimum nutrition domain’ was particularly emphasized in the Eastern Mediterranean Region (EMR) and the ‘learning, competence, education, skills and employability’ domain was particularly emphasized in the other three Regions (AFR, AMR, SEAR)) (Table 4). One may speculate that the greater emphasis on ‘good health and optimum nutrition’ by young people from EMR might reflect particularly strong concerns around mental health, conflict-related trauma, and healthcare access and, in turn, that this could be related to the substantial proportion of respondents from this Region living in countries affected by conflict. Similarly, one can speculate that a potential reason why young people from AFR and SEAR prioritized ‘learning, competence, education, skills, and employability’ (42.8% and 36.0%, respectively), may reflect currently unfulfilled aspirations related to economic mobility and structural barriers to schooling. Although still rarely mentioned, SEAR had the highest proportion of responses related to ‘agency and resilience’ (3.4%), including identity and purpose, which may reflect a greater desire for self-determination and youth empowerment.

Table 5 showed that young people identifying as male and female tended to prioritize the domain of ‘learning, competence, education, skills, and employability,’ reflecting aspirations tied to formal education and future economic mobility. In contrast, those identifying as agender, gender fluid, nonbinary, transwomen, transmen, and two-spirit—as well as those who selected ‘prefer not to say’—placed greater emphasis on ‘safety and a supportive environment.’ A larger proportion of this group also mentioned ‘wants’ related to ‘connectedness, positive values, and contribution to society,’ with this domain often ranking above education-related aspirations. These patterns suggest that for gender-diverse young people, well-being may be more closely associated with affirmation, inclusion, and protection than with institutional achievement. The data reveal both convergence and divergence: while male and female adolescents shared similar distributions across the domains related to health, education and safety, those identifying outside the binary tended to emphasize aspirations centered on agency, resilience, and social recognition. Their relatively lower prioritization of traditional domains such as employability may not reflect diminished ambition, but rather systemic exclusion or lack of representation in mainstream pathways.

However, these suggestions of potential underlying reasons for regional or gender-related variations are necessarily speculative, given that the WYPW survey captured brief, usually one-sentence responses and there was no possibility of exploring these more deeply. Complementary, in-depth qualitative research would be needed to unpack the reasons underlying each individual’s aspirations.

On the other hand, ‘wants’ related to all five domains of adolescent well-being were mentioned by at least 2000 respondents in all three age groups (10–14, 15–19, 20–24 years), in all four WHO Regions with adequate representation, and by both those who self-identified as women and men, which underscores its multi-component nature and the importance of multi-sectoral policies and programmes to achieve ‘what young people want’ to promote their well-being.

Preliminary findings from this survey informed the agenda of the Global Forum for Adolescents (GFA) in 2023 (PMNCH 2023a), the development of the Agenda for Action for Adolescents (PMNCH 2023b), and the commitments made by governments and others at the GFA (PMNCH 2023c), and continue to guide the work that PMNCH and partners, including key United Nations and nongovernmental agencies, are doing with and for adolescents and youth.

## Data Availability

The compiled survey responses are available on a PMNCH-hosted dashboard which can be accessed at: https://wypw.1point8b.org/en.

## Reflexivity statement

The author group is roughly matched by gender and spans a wide range of nationalities, country income groups, world regions and age.
